# Strategic universality in the making of global guidelines for mental health

**DOI:** 10.1177/13634615211068605

**Published:** 2022-01-19

**Authors:** China Mills

**Affiliations:** 1 4895 City University of London

**Keywords:** Global mental health, mhGAP, standardization, universality

## Abstract

Based on interviews with members of the Guideline Development Group (GDG) of the World Health Organization’s (WHO) Mental Health Gap Action Programme (mhGAP) Guidelines for Mental, Neurological and Substance Use Disorders, this article adds empirical depth to understanding the contingent and strategic nature of universality in relation to mental health. Differently from debating whether or not mental health is global, the article outlines the people, ideas, and processes involved in making it global. Thematic analysis of interviews carried out with nine (out of 21) members of the original mhGAP GDG identified six intersecting strategies that enable the construction of universality in global mental health (GMH): 1) processes and practices of assembling expertise; 2) decisions on what counts as evidence; 3) framing cultural relativism as nihilistic; 4) the delaying of complexity to prioritize action; 5) the narration of tensions as technical rather than epistemological; and 6) the ascription of messiness to local contexts rather than to processes of standardization. Interviews showed that differently from the public-facing consensus often presented in GMH, GDG members hold contrasting and contingent understandings of the nature of universality in relation to mental health diagnoses and interventions. Thus, the universality of mental health achieved through the mhGAP Guidelines is partial and temporary, requiring continuous (re)iteration. The article uses empirical data to show nuance, complexity, and multi-dimensionality where binary thinking sometimes dominates, and to make links across arguments ‘for’ and ‘against’ global mental health.

## Producing and legitimating mental health as global

Global mental health (GMH)—made up of an assemblage of actors, relationships, networks, ideas, practices, and technologies—has played a central role in constructing mental health as universal and putting the ‘global’ into mental health. In 2010, the World Health Organization (WHO) developed the Mental Health Gap Action Programme (mhGAP) Guidelines for Mental, Neurological and Substance Use Disorders (referred to hereafter as the mhGAP Guidelines). The mhGAP Guidelines are part of the WHO's Mental Health Gap Action Programme (mhGAP)—an attempt to standardize interventions to close the ‘treatment gap’ between need for mental health interventions and availability of care especially in low- and middle-income countries (LMICs). The Guidelines form the basis of a whole suite of mhGAP resources, including the mhGAP Intervention Guide (mhGAP-IG), Operational Guidelines, training materials, a smartphone app, and more.

The explicit global design of the Guidelines, their framing as a global standard (Patel et al., 2011), and that they provide the basis for derivative products key to scaling up mental health (WHO, 2009, 2011) makes them a significant focal point for research into the kinds of knowledge, techniques, and practices that construct and perform mental disorders as if they are universal. Focusing on the development of the mhGAP Guidelines gives insight into the processes through which universality is negotiated, enacted, and contested, and the “conceptual shifts and ruptures in the way universality is claimed” within GMH (Bemme & D’Souza, 2014, p. 856).

The construction of mental health as a global issue has been central within GMH messaging. For example, the Lancet Commission on Global Mental Health and Sustainable Development (2018) states that “mental health is a global public good,” a “universal human attribute and an indivisible component of overall health” (Patel et al., 2018, p. 1585). Key figures within the field state that:

the field of global mental health has arrived at something of a consensus: although there is ample evidence of differences in the expression of and responses to mental disorders, it is generally agreed that these “health conditions affect people in all cultures and societies”. (Cohen et al., 2013, p. 9)

Indeed, a key area of critique of GMH involves questioning the universality of mental health diagnoses and treatments, and their relevance to varying ‘local’ contexts (Das & Rao, 2012; Fernando, 2014). For example, Hailemariam and Pathare (2020) state that “it is time to reconsider whether work concerning global mental health is truly a global reflection” (p. 1012). Some of the concerns about mental health's universality come from those positioned ‘within’ GMH advocacy. For example, when asked, in 2014, about anticipated challenges for the field, Patel raised the issue of “continuing concern” over whether “common mental health problems” are “biomedical categories that have universal validity in all cultures” (Patel, 2014, p. 2). More recently, Saraceno (2020, p. 2) (Director of the WHO's Department of Mental Health and Substance Abuse from 2000 to 2010) asks whether GMH is “really global or rather Western” and questions the generic consensus around reducing the treatment gap. 

Debating the ‘global’ of mental health (while important) at times relies on an imagined incommensurable local/global binary, which obscures “emergent spaces, concepts, and fields of inquiry between and beyond” divides (Bemme & D’Souza, 2014, p. 852). In ethnographic research with GMH advocates, Bemme found that many talked about “the field's critical self-inspection” being stifled due to “the persistent fear” of “fuelling external critique,” which was felt to be “threatening” to the whole field (Bemme & Kirmayer, 2020, p. 11). This is helpful in understanding the disjuncture between a public facing portrayal of consensus, and simultaneous backstage tensions, about mental health's universality.

In interviewing members of the mhGAP Guideline Development Group, this research shows that those advocating for the “dominant view” in GMH are often aware of critique (Kienzler, 2019, p. 643), share concerns about the dominance of medicalization (Mills & Lacroix, 2019), and demonstrate the capacity “to easily change conceptual frameworks depending on their ‘plural affinities’ with different communities” (Bemme & D’Souza, 2014, p. 867). Yet those who develop interventions are not usually in a position to make “programming itself an object of analysis” (Li, 2007, p. 2). Thus, a key contribution of this research lies in taking a broader view—understanding the development of the mhGAP Guidelines as an epistemic object and foregrounding the reflections of those who do the work of making mental health global.

Differently from debating whether or not mental health is global, this article builds on research into the conditions of possibility that produce and legitimate mental health as global, i.e., focusing on how it is ‘done’ globally (through a specific set of guidelines) and what the framing of mental health as global ‘does’ (Mills & Hilberg, 2019). This further develops Bemme and D’Souza’s (2014) exploration of the configurations that made it possible for GMH to “go global” (p. 853).

A growing body of literature conceptualizes the field of GMH, and the unfolding of its various interventions, as an empirical object—tracing decision-making, scale-making, and evidence-making (see Bemme, 2019; Bemme & D’Souza, 2014; Cooper, 2015; Henckes, 2019; Kienzler, 2019; Lovell et al., 2019; Mills & Hilberg, 2019). Yet interdisciplinary scholarship into the standardization, classification, and development of medical protocols and guidelines (Bowker & Star, 2000; Lakoff, 2005; Timmermans & Berg, 1997, 2003a, 2003b) has rarely been used in relation to GMH (see Bemme, 2019; and Henckes, 2019 for rare examples). This article makes the argument that addressing this gap is significant in order to better document the nuanced strategic nature of universality in relation to mental health.

First the article explores how literature on standardization contributes to understanding the development of the mhGAP Guidelines. Next, the methodological process and analysis are detailed, followed by the findings and discussion.

## Standardization, guideline development, and the mhGAP Guidelines

Universality is “ambiguous and precarious” and is “contingently and collectively produced” (Timmermans & Berg, 1997, p. 277) as the result of the “historically situated, distributed work of a multitude of actors” (Timmermans & Berg, 1997, p. 288). Important historical conditions of possibility for the mhGAP Guidelines are the rise of evidence-based medicine (EBM) and particularly how EBM has shaped the WHO's development of guidelines, as well as key mental health classificatory systems, particularly the WHO’s International Classification of Diseases (ICD) (Mills & Hilberg, 2019).

Emphasis on mental health interventions as being evidence-based has been central to constructing mental health as global (Collins et al., 2011). EBM is itself an empirical object which has gained global currency, with historical work usually narrating it as developing from a need to “bring more certainty to clinical decision making,” made possible through linkages between the historically separate areas of epidemiology and medical research, to offer a more “systematized, scientific approach to the practice of medicine” (Sur & Dahm, 2011, p. 487). Where evidence that meets the criteria for EBM does not exist (which, as we will see, is quite common for mental health in LMICs) and in the movement from evidence to recommendations, expert judgement is mobilized to assess quality and applicability across contexts (Oxman et al., 2007).

EBM and systems of psychiatric classification, similar to the guidelines and protocols in which they are embedded, are the product of multiple local and political negotiations, making it important to better understand how “standard narratives that seem universal have been constructed” (Bowker & Star, 2000, p. 157). Systems of classification are a “significant site of political and ethical work” (Bowker & Star, 2000, p. 147) because every standard “valorizes some point of view and silences another” (p. 156), making them part of “the knowledge-power processes that inscribe and materialize” the world in particular ways (Haraway, 1997, p. 7), including inscribing and materializing mental health in some ways and not others (Cooper, 2015). The parameters of evidence established by EBM do not often include the lived and learned experience of patients and clinicians, and in rendering evidence ‘technical’ (Li, 2007) necessarily screen out the socio-political contexts in which people live and in which clinicians practice (Sur & Dahm, 2011, p. 487).

According to Lakoff (2005), standardization, through reduction of complexity, specificity, and locality, makes an asset transferable across different contexts—achieving ‘diagnostic liquidity.’ Diagnostic liquidity in mental health thus “requires consistent classificatory practice among doctors,” and reliance on techno-scientific objects (Lakoff, 2005, p. 68) that produce mental health diagnoses as “coherent and stable” (p. 77), and durable entities (p. 85) ‘with universal properties’ (p. 77). The production of guidelines is one of the central practices through which “the apparently universal validity of biomedical knowledge is materially and discursively forged via the standardization of practice across multiple domains” (Lakoff, 2005, pp. 66–7). Timmermans and Berg (2003b) analyze the development of medical guidelines as a “politics of standardisation in practice” (p. 21), where guidelines act as “coordinating devices” which structure and sequence practice (p. 77), enabling new “configurations of things and people” (p. 24). Here guidelines can be understood as the product of situated knowledge, emergent practices (Timmermans & Berg, 2003b, p. 22), and as “central mediators in the construction and reproduction of novel worlds” (Timmermans & Berg, 2003a, p. 108).

In this article, the mhGAP Guidelines (and derivative products) are understood as techno-scientific objects (Timmermans & Berg, 1997), whose “network of production and stabilization” (Lakoff, 2005, p. 77) is explored in order to shed light on the emergent and negotiated production of GMH. This article sees the mhGAP Guidelines as involving material, institutional, and discursive practices that aim to (strategically and contingently) produce universality in mental health. The research draws upon two central ideas. One is Bemme’s (2019) discussion of “contingent universals”—in GMH—“concepts that are true and measurable until they stop working in the field, or until the parameters of ‘what works’ shift to a new iteration” (p. 575). Analysis of contingent universals must be situated within “a broader lineage of how the ‘universal’ has been crafted, mobilized and critiqued” (Bemme, 2018, p. 149). The second is what Voronka (2016) (a scholar who identifies as having lived experience of the mental health system) calls ‘strategic essentialism.’ Voronka (2016) explores how the universalization of identity categories such as user, survivor, and/or lived experience can risk conflating and treating as homogenous distinct “ideological and conceptual explanatory models” (p. 196). Her work is adapted here to think through the strategic nature of the crafting of universality within GMH.

## Method

The mhGAP Guidelines are multiple—practiced and *done* (talked about and used) differently in different contexts—and these differences are analytically important. This article is interested in the overlap and contrast between official documentation about the methodological process of making the mhGAP Guidelines and how this process is narrated by those involved in their design. A useful approach to exploring insider critique within GMH is to engage in ‘studying up,’ using sociological and ethnographic methods of researching decision-making through ‘elite interviewing’ and participation at high-level events and meetings (Maes, 2015). Between July and December 2018, 19 interviews were carried out in total: nine (out of 21) with members of the original mhGAP-IG Guideline Development Group (GDG) (discussed below); and 10 with individuals who have experience implementing the mhGAP Guidelines through mhGAP-IG in LMICs. This article largely focuses on data from the former, while the latter are documented in Mills and Lacroix (2019). Over half the interviews were carried out online because participants were based in multiple geographical locations. An interview protocol and semi-structured questions were developed as a guide. Interviews with GDG members were carried out by the author of this article and lasted 30–90 min. In total, 18 people (out of 21 original members of the GDG) were contacted by email for interview; 12 agreed to participate and nine proceeded to interview. Sampling occurred through snowballing—a continuous process where participants were asked to suggest others who should be contacted for interview.

The interview protocol asked about the often “behind-the-scenes boring background processes” (Bowker & Star, 2000, p. 156) of the production of the mhGAP Guidelines, aiming to foreground the “routine and mundane workings [that] do not find their way into institutional history” (Kienzler, 2019, p. 638) yet are important sites of knowledge production. The initial design of the mhGAP Guidelines occurred in 2008, meaning that the focus of this article is on the different ways they are talked about retrospectively by those who designed them—significant because these early assumptions are written into what is now being used globally. Yet memory, and likely many other influencing factors, impact the findings, and indeed participants did remember the process differently. To address important limitations in the methodological approach, triangulation of data was achieved through close reading of WHO official literature, including mhGAP methods papers (discussed in the analysis), and participant observation (by the author) of, and attendance at, GMH events (see Mills & Lacroix, 2019, for details) (also see below in eliciting different perspectives during early coding). A final draft of this article was also sent to some of the participants and key figures in the development of the Guidelines for fact-checking. Attempts were made to contact the WHO mhGAP team but these were unfortunately unsuccessful. Yet the underlying approach of this research is constructionist, meaning that narratives are themselves emergent social practices and products of negotiation (De Fina & Georgakopoulou, 2008), therefore the article does not evaluate the truth claims of people's different understandings.

### Ethics

Ethical approval was granted from the Principal Investigator's university at the time of the research (University of Sheffield); and from the IRB at Sangath, Goa, India. All participants were sent an information sheet prior to the interview; all signed a consent form; and all were given opportunities to ask questions both before and after the interviews. As a quality check, participants were asked if they felt any questions were missing that should have been asked.

### Thematic analysis

All interviews were audio-recorded with participant consent. Recordings were transcribed verbatim. All transcripts were checked against the recording by the principal investigator.

During the interview, the interviewer kept written notes, which were later used to inform the analysis. The interview data were analyzed using thematic analysis, understood here as a diverse set of “theoretically flexible” approaches, which provide “robust, systematic framework[s] for coding qualitative data” through identifying themes (patterns of meaning) across a dataset (Braun & Clarke, 2014, pp. 1–2). The thematic analysis was informed by Braun and Clarke’s (2014) six-stage process but was adapted slightly to include further cross-checking of early coding and reviewing of initial themes. The data was coded by hand to enable deep familiarization, and formulation of themes was an “active process of pattern formation and identification,” in contrast to the assumption that findings exist in the data, waiting to be discovered through analysis (Clarke et al., 2019, p. 18). Researcher interpretation was “integral to the process of analysis” (Clarke et al., 2019, p. 6) and not a source of bias to be minimized. Positionality played an important shaping role in the research given that the lead researcher and author of this article has been researching GMH since 2009 and was known in some capacity to most of the participants. Latent coding enabled attention to be paid to the assumptions underlying the data, while a constructionist approach to coding focused attention on how different versions of reality are created by the data.

Alongside thematic analysis of the interview data, an ethnographic stance (Li, 2007) was taken in analyzing ‘official’ documents relating to the production of the mhGAP Guidelines (all available online through the WHO mhGAP Evidence Resource Centre; see below). The method of looking at both official documents and interview data together is not to privilege the truth claims of one over the other but to show that even where transparency is emphasized by the WHO in guideline development and detailed documentation publicly available, the resulting account is always partial (as is my account in this article). By focusing interviews on the “behind-the-scenes” mundane processes (Bowker & Star, 2000, p. 156) in the production of the mhGAP Guidelines, the findings illuminate perspectives that may be missing from both the official documentation process and the consensus presented in official GMH literature (Dua et al., 2011; WHO, 2015).

After first coding, the main themes were presented to an invited group, including some of the original participants, service user and psychiatric survivor researchers, and practitioners, at an advanced studies seminar on standardization in mental health.^1^ This helped to assure quality by encouraging reflection and increasing the rigor of analysis by exposing the dataset to different perspectives, and to increase complexity and depth of engagement with the data (Clarke et al., 2019). The findings are presented below; direct quotes are identified by a specific number given to each participant (R1–9).

## Findings

Developed through latent and constructionist approaches to coding, this research identified six intersecting strategies that enable the construction, and show the strategic nature, of universality in GMH. These strategies include processes and practices of assembling expertise, and decisions on what counts as evidence; alongside framing cultural relativism as nihilistic; the delaying of complexity to prioritize action; the narration of tensions as technical rather than epistemological; and the ascription of messiness to local contexts rather than to processes of standardization. For ease of reading, each of these themes will be discussed in turn, yet the intersections between the themes are themselves key in constructing universality in GMH.

### Assembling the experts and creating consensus

A WHO (2012) guideline contains clinical, public health, or policy “recommendations about health interventions” to inform decision-making (p. 1). The WHO has a standardized approach to the development of evidence-based guidelines, as outlined in its *Handbook for Guideline Development* (2012). The Handbook stipulates that new guidelines, such as the mhGAP Guidelines, must be developed following a set of key principles, and that the development process should be transparent and multidisciplinary (WHO, 2012).

The ‘official’ methodological process of developing the original mhGAP guidelines is outlined in a number of publications (see Dua et al., 2011) and by the WHO mhGAP Evidence Resource Centre (WHO, 2009). The WHO (2009) explain the mhGAP Guideline Process as starting with identification, and creation of a global network, of ‘experts.’ Emphasis was given to multidisciplinary expertise, which included ‘caregivers’ but does not name inclusion of people with lived expertise. (It is worth noting that this may change in future iterations, given that the principle of the participation of those with lived experience is emphasized in the WHO Mental Health Action Plan 2013—now extended to 2030). From this initial network, a subset of experts then formed the GDG, which was “convened to advise on the content and process, interpretation of evidence, to formulate and finalize the recommendations” (WHO, 2009, p. 3). The formation of the GDG was led by Shekhar Saxena (then Director of the WHO's Department of Mental Health and Substance Abuse). While the GDG is the focus of this article, the “guideline development group was supported by a much larger number of people” (R7).

The WHO (2009, p. 3) states that “adequate regional and gender representation were identified” as important in the formation of the GDG. In my interviews with GDG members, there were differences in how members perceived the group—with some seeing it as “representing a diverse range of perspectives” (R6) and others seeing it as a “very international” yet “homogenous group of people,” a group of “mates who wanted to change psychiatry,” “with no representation from mental health service users” (R8). Differing perceptions of diversity within the group are interesting given that of the 21 members making up the original GDG, there were no people who publicly identify as service users, psychiatric survivors, or people with psychosocial disability; five of the 21 are female; and 18 of the 21 are psychiatrists and/or Professors of Psychiatry (with the remaining three made up of two Psychologists and one Neurologist) (although these formal job titles do not detail the wider array of shifting positionalities and experience that GDG members may occupy).

This is consistent with Oxman et al.’s (2007) findings, from their interviews with departmental managers within the WHO, that those deemed experts in the making of recommendations are usually those with knowledge in a specific area and not “representatives of those who will have to live with the recommendations” (p. 1887). Interestingly, when asked about the initial selection of the GDG, none of the participants could give details beyond ensuring that members met criteria for no conflict of interest (COI). This is a stipulation of the WHO's *Handbook for Guideline Development* (2012) (see Chapter 4), yet participants talked about it as an outcome of group consensus, saying we “wanted to not be seen as having our hands dirty from critics … didn’t want to be seen as being conflicted” (R8). Thus, concerns about criticism shaped guideline development: “critical voices from outside [the GDG] thought that people inside hadn’t thought about dangers, whereas people inside felt checks and balances had been put in place” (R8).

Following the formation of the international network of experts came the framing of “priority conditions”—"depression, schizophrenia and other psychotic disorders (including bipolar disorder), suicide prevention, epilepsy, dementia, disorders due to use of alcohol and illicit drugs, and mental disorders in children” (WHO, 2009, p. 2). These conditions were “identified on the basis of high mortality and morbidity, high economic costs, or association with violation of human rights” (WHO, 2009, p. 2). Once the priority conditions were identified, the formulation of scoping questions began, in consultation with an international expert panel using the PICO framework (Population, Intervention, Comparator, Outcome, Time) (WHO, 2009, p. 4). One participant remembered “that there was quite a bit of discussion about these scoping questions, and around the focus of these recommendations” (R4). The groups were organized in a way to achieve consensus, meaning the experts were “divided into small groups, and then there was kind of a plenary discussion after that to reach a consensus,” which “worked reasonably well, because of course it makes a difference if you have a discussion with 20 people or with five” (R4). Here the format of discussions is structured to foster consensus. According to published literature on the methodology of the mhGAP Guidelines, once formulated, the scoping questions were then followed by “systematic reviews of the best available evidence” in order to develop evidence profiles for each priority condition (Dua et al., 2011, p. 2). In 2015, a ‘WHO mhGAP Guideline Update’ was published and used to inform development of the mhGAP-IG version 2 (2016).

### Assembling the evidence

Classification of evidence in the making of medical guidelines is a “situated process” where evidence “is an emergent category produced during the developmental process of each guideline” (Knaapen, 2013, p. 684). The WHO's strong emphasis on evidence-based guidelines (Dua et al., 2011) raises important questions over what counts as evidence in GMH. Participants talked about the GDG making “a choice of giving priority to the results of systematic reviews” (R4) for shaping the recommendations made within the Guidelines—following the WHO’s (2012) *Handbook for Guideline Development*. This process exposed a lack of systematic reviews for GMH (R6), for which “new systematic reviews were commissioned” (WHO, 2009, p. 5)—a process recommended by the WHO (2012). One participant said they “were actually surprised not at the evidence but at the lack of evidence” (R7). Similarly, the official literature identifies a number of challenges in creating the mhGAP Guidelines, including no or very poor-quality evidence “insufficient to make any recommendation,” for example, as was the case for some psychosocial interventions (Dua et al., 2011, p. 2).

Where evidence existed or was commissioned, it was then evaluated in “consideration of values, preferences, and feasibility issues from an international perspective” (Dua et al., 2011, p. 2). The evidence was graded using the Grading of Recommendations Assessment, Development and Evaluation (GRADE) approach, which synthesizes the scientific evidence on the effectiveness of clinical interventions (Dua et al., 2011; WHO, 2009, 2015) (see Barbui et al., 2015 on the use of GRADE in updating the mhGAP recommendations). GRADE methodology includes only data that meet specific pre-determined scientific criteria (from systematic reviews and randomized control trials (RCTs)), resulting in the exclusion of qualitative data and thus of experiences not easily captured by standardization (Cooper, 2015). Interview data show that many GDG members were aware of the potential pitfalls of relying on randomized evidence, including overlooking what might be helpful but is not captured by randomized design (R4 and R7). Despite the Guidelines containing multiple recommendations for psychosocial interventions, some participants raised concerns that the emphasis on randomized evidence (of which there is more for drugs than psychosocial support given pharmaceutical industry-sponsored trials) led to a pharmaceutical skew to the Guidelines. For example, one participant describes how this has led to criticism from “people saying actually what you are implementing is more use of medicines, with a risk of medicalizing social problems” (R4).

### The nihilism of cultural relativism and doing mental health in ‘the meantime’

A particular issue that was described as eliciting discussion amongst the GDG was about the applicability of evidence from HICs to LMIC contexts. One participant summarizes that one of the “major limitations of the whole process is that eighty or ninety percent of all the evidence upon which the recommendations were made were from high-income countries,” and yet was going to be applied in LMICs (R6). Yet the GDG decided early on that they would not disregard HIC evidence.

This decision reflected deeper concerns among the GDG about the cultural relativity of diagnoses and treatments. For those interviewed, cultural relativism was understood to mean that “social and cultural practices are so important that you couldn’t do anything” and that “evidence generated in one context cannot be applied in any other context” (R1). This was understood as a “prevailing nihilistic view that had really been very destructive actually,” leading to a “paralysis in the mental health sector” over the past 40 years (R1). The mhGAP Guidelines were thus imagined as having both political and cultural value in shifting this nihilism by showing “you could have certain common rules that can be applied across contexts” (R1), and by moving “mental health into an equal position with physical health” (R3). This perceived nihilism is thus a condition of possibility for the Guidelines, evident in the way some participants evoked a time “before mhGAP” where healthcare systems are described as “doing nothing” or as enacting harm, and meaning that the “greatest contribution” of the Guidelines was to “make systems feel there was something they could do” (R1). The idea of ‘nothing before mhGAP’ is consistent with GMH's emphasis on both scarcity and urgency (Bemme & Kirmayer, 2020).

The mhGAP Guidelines are framed as being ‘urgently needed’ (Dua et al., 2011), and constructed as enabling action, as opposed to the supposed nihilism of cultural relativism. The conversion of evidence into recommendations was also impacted by decisions about public health benefit (R7), where the assumed cost (to public health) of inaction (i.e. having no guideline) was weighed against potential risks of action (i.e., recommendations that were unlikely to be able to be effectively delivered). These assumptions are evident in how participants recognize complexity yet delay or defer the implications of this in order to prioritize action.

Participants openly acknowledged potential problems with the Guidelines, explaining that it is still “a fairly crude measure, but it's probably the best we can do for now” (R3); “I feel that the decisions made were the best decisions that the group at this time could have made for public gain” (R7); “any first version is not going to be perfect by a long way” (R6); and that “there's a whole complexity to mental health care that cannot be captured in a 100 page guideline” (R1). In this way, GMH works in the “meantime” between “the critical view of what could be and the pragmatic sense of what is” (McKay, 2018, p. 198). Key to doing mental health in the meantime is the process of rendering mental health technical.

### Rendering mental health technical

When asked about any tensions within the group, participants’ accounts centered more on technicalities than on epistemologies, such as arguments over the use of certain drugs “in the field” as being “too dangerous” (R8). According to R1, the Guidelines “were always intended just to be a technical tool.” Despite the design of the mhGAP Guidelines assuming the universality of diagnostic categories and interventions, universality was discussed by participants as differing between conditions and between interventions, and was thus portrayed as contested, emergent, and strategic (quite different from the consensus often portrayed in much of the GMH and WHO literature; see Cohen et al., 2013). Those interviewed held differing understandings of the universality of different diagnoses and interventions. For example, GDG members spoke of schizophrenia and psychosis as more universal and transferable across cultures than mood disorders, whose universality was described as contested and negotiable. As with diagnoses, the interview data suggest that some interventions have achieved different levels of durability and universality, making some more ‘liquid’ than others. For example, while pharmacological interventions were seen as universal, this wasn’t seen to extend to psychological interventions, such as Cognitive Behavioural Therapy (CBT), which while projected as “culturally neutral” were described as “not universal” (R2).

Yet these differing ideas about the universality of mental health were not recalled by participants as points of discussion or tension in the making of the mhGAP-IG. This may be because universality was foundational to rendering GMH technical through development of the Guidelines. Instead, participants recall of tensions centered on technical issues rather than around the epistemic infrastructure and epistemology of mental health's universality, while problematization occurred more for the contexts and means of implementation.

### Messiness of context, not of design

The assumption that the Guidelines would require adaptation for use in different contexts was thought by some members of the GDG to be part of their design (R4), yet processes of local adaption were said to have “varied enormously” ranging from “major restructuring” to translation (R3). Conceptualizations of ‘context’ thus emerged as an issue in the implementation of the Guidelines but less so in discussion of the contextual factors shaping their design (Mills & Lacroix, 2019).

Only one participant (R2) problematized the design of the Guidelines in relation to implementation, explaining that the universal design of the mhGAP Guidelines and their underlying assumptions of mental health as global leave little to be adapted. This is because the Guidelines are made possible by the assumption that “symptoms are universal in nature” and are thus designed to be “context neutral” (R2). Thus, the Guidelines reflect a strong desire for universality—part of a project to “create universal things,” whereby “cultural context becomes like footnotes” (R2).

Others shared concerns that:

one of the limitations of mhGAP is that it's very much a kind of disorder-based construct. And the tricky thing with the way in which people present in primary healthcare settings is that they don’t knock on the door and say “hello, I’m depressed” … it's very messy actually, in the real world of primary care clinics. (R3)

Here messiness is ascribed to local contexts of primary healthcare, and not to the messy process of designing and constructing guidelines, nor to the messy work of standardization. Context seemed particularly key in participants’ understandings of how the Guidelines, which include more psychosocial than medical recommendations, may contribute to medicalization less so by design than through their interaction in specific contexts:

when guidelines present both psychosocial and social interventions as well as drug interventions what de facto happens, is the drug interventions are the one that have the most traction. At the end of the day it could be that the prescribing of medicines is the easiest way of at least doing something. (R1)

It seems that despite the design of guidelines to include psychosocial interventions, drug treatments may prevail because of the context in which guidelines are enacted—contexts of over-stretched health systems and workforces, which privilege medical solutions to mental health.

## Discussion and conclusion

The mhGAP Guidelines are an example of a key techno-scientific object that helps produce the universality of mental health (Lakoff, 2005, p. 68). Yet thematic analysis of interviews with decision-makers involved in the design of the Guidelines shows that differently from the public-facing consensus often presented in GMH (Cohen et al., 2013), GDG members hold contrasting and contingent understandings of the nature of universality in relation to mental health diagnoses and interventions.

The universality achieved through the mhGAP Guidelines is thus partial and temporary—consistent with the ‘contingent universals’ discussed by Bemme (2019), and with the WHO's conceptualization of the Guidelines as iterative and in need of constant revision. This contingent and iterative approach shows the inherently unstable universality of mental health. At first, I conceptualized this process as a form of ‘negotiated universality.’ Yet not everyone gets a place at the negotiation table—and people with lived experience (for example, who identify as people with psychosocial disability or diversity, service users, psychiatric survivors), and user/survivor epistemologies (Rose, 2017), are often missing from GMH decision-making. As mentioned earlier, this may change given the emphasis in the WHO's (now extended to 2030) Mental Health Action plan on user participation—something we may see as the new GDG is being put together for the five-yearly review process of the Guidelines (which is happening as I write). This raises wider issues about the role of guidelines in GMH, including assumptions about who guides and who is to be guided—and whose expertise is made to count.

Nor is the universality achieved through the Guidelines always contested, at least not openly. It may be that the public-facing construction of consensus in GMH is a response to real and perceived external critique (which may be perceived as stifling internal critique from being discussed openly) (Bemme, 2018). Given the emphasis from GDG members on the Guidelines as having cultural and political value, and as serving an important purpose in the ‘meantime’ (McKay, 2018) of GMH, perhaps the universality achieved through the Guidelines is better understood as ‘strategic,’ enacting what Voronka (2016) calls ‘strategic essentialism.’ For example, the GDG hold differing views about the universality of mental health yet employ a range of strategies (discussed in the analysis) which flatten out the differing perspectives of the GDG as a political move to enable action—to get something done—i.e., in this case to produce global guidelines framed as key to closing the treatment gap and alleviating suffering.

This article has traced some of the intersecting strategies through which strategic universality is achieved in GMH through focusing on one set of global guidelines. Some of these strategies are processual—evident in the organization of the GDG, the assembling of experts, the format of discussions structured to foster consensus (for example, in small groups), and decision-making on what counts as evidence. Other strategies included framing cultural relativism as nihilistic; the delaying of complexity to prioritize action; the narration of tensions as technical rather than epistemological; and the ascription of messiness to local contexts rather than to processes of standardization. Thus, the strategic universality of GMH depends upon a process of rendering mental health technical—described by Li (2007, p. 10) as the construction of a “problem” in “technical non-political terms amenable to technical (and non-political) interventions.” This process is always partial, meaning that the questions that “experts exclude, misrecognize, or attempt to contain do not go away” (Li, 2007, p. 10)—just as questions of universality resurfaced in the interviews with GDG members. While critical research has paid attention to what is screened out when GMH is rendered technical (including experiential and local knowledge) (such as Cooper, 2015), less has explored processes of ‘rendering technical’ (Li, 2007), making this a fruitful area for future research.

Exploring what it means for mental health to be framed as a “globally conceived object of care,” Bemme and Kirmayer (2020, p. 6) suggest that the “global” in GMH is an “organizational project” laboring to render mental health amenable to global technical interventions and made possible through the mobilization of “scarcity” (epitomized by the “treatment gap” that the mhGAP Guidelines seek to close) (pp. 4–5). Here mental health becomes whatever can be simplified and rendered technical—i.e., what can be taught and delivered in a set number of sessions over a specific time period using as few resources as possible (Bemme & Kirmayer, 2020). Intervention protocols, such as the mhGAP Guidelines, designed for low-resource settings, thus constitute “testing grounds for new and simplified psychiatric classifications and models of care,” which, when translated into scientific evidence, are “rendered mobile” (Bemme & Kirmayer, 2020, p. 7).

Once made technical, the mhGAP Guidelines intersect with local health infrastructures (conceptualized as being ‘messy’), and it is in this interaction that some participants saw potential for pharmacological recommendations to dominate. It remains unclear then if and/or how medicalization is embedded into the design of the Guidelines, given that the recommendations include psychosocial support. What seems clearer is how local realities of implementation (i.e., availability of pharmaceuticals, and their perceived simplicity and universality compared to psychosocial interventions) may enable pharmaceutical interventions to dominate treatment recommendations, even when they appear alongside psychosocial interventions in the Guidelines. A clue to understanding why drug treatments may dominate in guidelines where psychosocial interventions are also recommended is given by R2—that drug treatments, differently from other interventions, are already assumed to be universal, and thus contexts of over-stretched health systems and workforces may privilege medical solutions to mental health. This privileging is itself the product of political and economic factors (e.g., availability and pricing of drugs, intellectual property and patenting regimes, categorization of specific drugs as ‘essential medicines’ etc.) that are often screened out as mental health is rendered technical (Li, 2007).

Throughout the interviews, participants showed nuanced and complex understandings, some of which align with social scientific critiques of GMH (Kienzler, 2019; Timmermans & Berg, 2003a). Yet participants’ acknowledgment of critique and complexity was often accompanied by the (temporary) prioritizing of a pragmatic approach to act now and thus to delay attending to complexity. Here the “the prescribing of medicines is the easiest way of at least doing something” (R4), suggesting that delayed complexity in favor of urgent action, i.e., doing mental health in the ‘meantime’ through rendering it technical, intersects with decisions about evidence and local health infrastructures.

While this research makes an important contribution to the literature on the construction of universality within GMH, the research has a number of limitations. For example, it did not examine in detail how the mhGAP Guidelines are used in practice in different contexts; it did not explore the larger epistemic infrastructure in which the Guidelines are embedded; nor, crucially, did it look at the impact its use has for those whose lives and subjectivities may come to be shaped by its diagnoses and recommendations. The data rely on retrospective accounts of the production of the Guidelines, and the small number of participants makes wider generalization impossible.

Despite these limitations, this article suggests that the universality of mental disorder is contingent and strategic, and that the topic of strategic universality, and the “strategic essentialism” (Voronka, 2016) and “contingent universals” (Bemme, 2018, 2019) from which it draws, may provide an important emergent field of inquiry to work with and across different perspectives, moving away from the demand of consensus. In applying Voronka's conceptual work to a different context, this article also shows the importance of user-survivor research and experiential knowledge derived from lived experience for analyzing, understanding, and transforming GMH (while simultaneously remaining aware that ‘lived experience’ can itself be deployed in essentializing and strategic ways). Critical approaches may achieve more nuance then in not taking at face value the public-facing consensus portrayed by some GMH advocates, and instead exploring the flattening or (temporary) deferral of epistemological differences that goes into producing consensus in the ‘meantime’ of GMH. The inherently unstable universality of mental health allows attention to be turned to the conditions and contexts that produce the perception held by some within GMH that strategic universality is required or desirable, shifting focus onto the labor that goes into making mental health global.

## Notes

1. The advanced studies seminar was titled “Making Mental Health Count: Quantifying and Standardising Mental Distress,” and took place on May 8, at St Catherine's, University of Oxford.
